# Sealing of anodized magnesium alloy AZ31 with MgAl layered double hydroxides layers†

**DOI:** 10.1039/c7ra11683g

**Published:** 2018-01-09

**Authors:** Gen Zhang, Liang Wu, Aitao Tang, Bo Weng, Andrej Atrens, Shida Ma, Lei Liu, Fusheng Pan

**Affiliations:** College of Materials Science and Engineering, Chongqing University Chongqing 400044 China wuliang@cqu.edu.cn tat@cqu.edu.cn +86 17783101968 +86 23 65106121; National Engineering Research Center for Magnesium Alloys, Chongqing University Chongqing 400044 China; Chongqing Key Lab for Advanced Materials & Clean Energies of Technologies, Institute for Clean Energy and Advanced Materials, Southwest University Chongqing 400715 China; School of Mechanical and Mining Engineering, The University of Queensland Brisbane Qld 4072 Australia

## Abstract

In this work, anodized magnesium alloy AZ31 with and without boiling water sealing was pre-prepared, and then MgAl-layered double hydroxide (LDH) films were fabricated on it through hydrothermal chemical conversion of the pre-prepared anodic layer. The morphology, structure, and composition of the films were characterized by XRD, SEM, EDS, FT-IR, XPS and GDOES. It was found that the porosity of the films was reduced after *in situ* fabrication of the LDHs. The effects of boiling water sealing treatment on the anodized substrate were also discussed. Moreover, the polarization curve, EIS, and immersion tests showed that LDHs fabricated on the anodized substrate with boiling water sealing treatment exhibited a significant long period of protection for the substrate.

## Abstract 2

In this work anodized magnesium alloy AZ31 with and without boiling water sealing was pre-prepared, and then MgAl-layered double hydroxide (LDH) films were fabricated on it through hydrothermal chemical conversion of the pre-prepared anodic layer.

## Graphical abstract

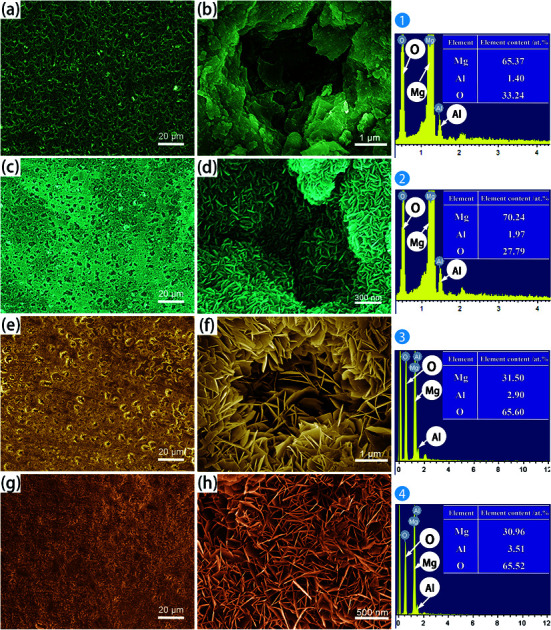

## 1. Introduction

Magnesium alloys, as the lightest structural metal, are increasingly being considered as an alternative to other metallic structural engineering materials. Unfortunately, their highly reactive nature and low corrosion resistance inhibit their wide scale use in many applications.^1,2^ To date, a variety of methods have been proposed and developed to protect magnesium alloys from corrosion. Examples including, purification of magnesium alloys, homogenization of microstructure and addition of rare earth elements^3–5^ are metallurgical methods to improve the corrosion resistance of Mg-based alloys. However, protection against general corrosion and galvanic corrosion remains a great challenge. Therefore, surface treatments including anodizing, conversion films, vapor deposition, flame or plasma spraying are considered to be other approaches to improve corrosion resistance.^6–8^

As an industrial technology for surface protection, the anodizing process has been successfully used over many decades.^9^ Nevertheless, the structures of anodic films consist of an inner thin compact layer and an outer thick porous layer. Although the porous structure is conducive to be coloured by organic dyes or inorganic pigments, porous anodic layers are defects, which reduce the corrosion resistance or even accelerate the corrosion damage of the substrate.^10^ Consequently, a sealing treatment is a necessary step after anodizing to enhance corrosion protection. The most common sealing treatments are conducted using boiling water, silicates, sol–gel and polymer coatings.^11–14^

Recently, conversion films also have been widely studied since they are inexpensive and simple.^9^ Layered double hydroxides (LDHs) as a chemical conversion film have been developed to improve the corrosion resistance of the metallic substrate. LDHs are a class of two-dimensional nanostructured anionic clays, with a structure that can be described as a brucite-like layer, and by the general formula of [M(ii)_1−*x*_M(iii)_*x*_(OH)_2_][A^*n*−^]_*x*/*n*_·*m*H_2_O, where M(ii) and M(iii) are divalent and trivalent metal cations, respectively, and A^*n*−^ are interlayer charge-compensating anions. Based on their ion-exchange capability, the LDHs can act as nanotraps that release interlayer anions and store corrosion-relevant anions such as chlorides. As a result, LDHs can delay the diffusion of ‘aggressive’ ions to the metallic substrate surface.^15,16^

Previous studies^15–18^ used coprecipitation to prepare LDHs. One major disadvantage of coprecipitation is the poor adhesion between the film and the substrate compared to other methods. Moreover, this methodology is complex, time consuming, poorly crystallized and produces large amounts of wastes.^19,20^ The steam^7,21,22^ is also a common method to synthesize LDHs, but is accompanied with the low content of LDHs, even with no LDH ingredient.

Compared to these methods, the *in situ* method directly grows films on the metal substrate. This can also considerably improve the adherence to the substrate and the mechanical stability of the film.^23^ Furthermore, although the film does not consist of a single LDH phase, the amount of the impurity phase is low. Tedim and colleagues^24–26^ prepared ZnAl-LDHs on aluminium alloys using a Zn^2+^ containing aqueous solution. They found that the surface of the metal was covered by a thin film separated by micro-metre sized islands where the LDHs were concentrated. They demonstrated that a relatively high dissolution of Al^3+^, which came from the intermetallics, promoted the preferential growth of LDHs in the area of the intermetallic phases. Nevertheless, the surface could not be completely covered by LDHs because of the island structure, which was not good for the long-term protection of the metallic substrate. Wu *et al.*^27^ reported a electrochemical deposition method for preparing ZnAl-LDHs on magnesium alloys. Zhou *et al.*^28^ developed a ZnAl-LDHs nitrate on the magnesium alloy by immersion of Mg sheets in Zn and Al containing solution, and was then intercalated with Cl^−^ and VO_3_^−^ respectively. They found that the concentration gradient wall of chloride anions in LDHs chloride films successfully delayed the diffusion of aggressive chloride ions to the magnesium alloy surface and LDHs vanadate films not only absorbed aggressive chloride ions but also released vanadate anions in solution.

In several recent studies, LDH films were prepared on an anodized aluminium alloy rather than directly on the bare metallic substrate.^29–31^ On the one hand, the pores were sealed by LDHs, which were formed on the anodic layers. On the other hand, the thin compact inner layer of the anodic film, and the LDH films with the high density, could stop ‘aggressive’ ions from reaching the metallic substrate. Furthermore, Li^30^ and Kuznetsov^31^ studied the difference of LDH films formed on anodized aluminium alloy with and without boiling water sealing. They found that the growth of LDHs was greatly influenced by the boiling water sealing treatment on the anodized aluminium alloy. A more compact arrangement of LDH nanosheets could be gained by this method. However, the mechanism of the boiling water sealing for anodized aluminium alloy on the growth of LDHs was not thoroughly analysed. And, there are few publications reporting the formation of LDHs on anodized magnesium alloys. Moreover, Chen *et al.*^32^ believed that the amount of Al, which was dissolved from the Mg alloys of low Al content, such as AZ31, was far from sufficient for the formation of MgAl-LDH films. Thus, it is necessary to add Al containing compounds to the precursor solution for the synthesis of LDHs. Nevertheless, such metal solution is acidic due to the hydrolysis of the metal salt, and the pH of the solution must be adjusted for the synthesis of the LDHs.

In this study, the magnesium alloy AZ31 was anodized in a solution of NaOH and NaAlO_2_. The Al could enter into the anodic films from the anodizing solution. The anodic films, including enough Mg and Al mixed oxide, could act as the internal source of divalent and trivalent metal cations to prepare the LDHs. LDHs were prepared in deionized water only without adding extra salts in this novel method. And, the influence of boiling water sealing treatment on the growth of LDHs was also studied. The structure, morphology and corrosion behavior of the LDH films were investigated by physical, chemical and electrochemical methods.

## 2. Experimental methods

### 2.1 Materials

The cast magnesium alloy AZ31 was used as the substrate, with the following nominal composition in wt%: Al 2.5–3.5, Zn 0.6–1.3, Mn 0.2–1, Ca 0.04, Si 0.1, Cu 0.05, and balance Mg. All reagents were analytically pure and were used as raw materials without further purification. Deionized water was used as a solvent.

### 2.2 Anodizing and sealing

Samples of 10 × 10 × 5 mm and 20 × 20 × 5 mm size were ground to 2000 grit SiC paper, then were anodized in a solution of 7.14 g L^−1^ NaOH and 4 g L^−1^ NaAlO_2_ for 30 min with an applied voltage of 20 V, ultrasonically cleaned in ethyl alcohol for 5 min, and dried under a steam of air.

After anodizing, some samples were sealed in boiling water at atmospheric pressure for 20 min. Similarly, some samples were dried under a steam of air.

### 2.3 Preparation of LDHs

To prepare MgAl-LDHs, the samples were immersed vertically in deionized water and heated in a Teflon-lined autoclave at 398 K for 12 h. This method does not introduce any kinds of metal salts. The preparation conditions of the different samples are summarized in Table 1. For clarity of discussion, anodized substrates without and with boiling water sealing were denoted as A and AS respectively. Furthermore, LDHs fabricated on anodized substrates with and without boiling water sealing were denoted as A-LDH and AS-LDH respectively.

**Table 1:** The preparation conditions of the samples

Sample	Anodizing	Pre-treatment	Post-treatment
		Sealing	Preparation of LDHs
A	+	−	−
AS	+	+	−
A-LDH	+	−	+
AS-LDH	+	+	+

### 2.4 Characterization

The surface and cross-sectional morphologies of the as-prepared samples were observed using a field-emission scanning electron microscope (FE-SEM; Nova 400 FEI, USA). For cross-sectional examinations, sections of the samples were generated by ultramicrotomy (UC; Leica EM UC7, Germany) using a diamond knife. The chemical composition was investigated using energy dispersive spectra (EDS; INCA Energy 350 Oxford, UK) and X-ray photoelectron spectroscopy (XPS; ESCALAB 250Xi, USA) with Al K_α_ radiation (1486.6 eV). The analyzed area for XPS was about 25 mm^2^ at the center of the surface of samples. Fourier-transform infrared (FT-IR; Nicolet IS5 Thermo Scientific, USA) attenuated total reflection spectroscopy (ATR) was obtained in the wavenumber range of 4000–400 cm^−1^. Glow discharge optical emission spectroscopy (GDOES; GD Profile 2, French) depth profile analysis of the films was carried out at a pressure of 700 Pa and at power of 40 W. The structures of the obtained LDH films were examined using an X-ray diffractometer (XRD; D/Max 2500X Rigaku, Japan) at a glancing angle of 1.5° using a Cu target (40 kV, 150 mA), within the range of 2*θ* = 5–80° and at a scanning rate of 4° min^−1^.

The electrochemical impedance spectra (EIS) and potentiodynamic polarization curves (PDP) were obtained using a CIMPS-2 Zahner system. A classical three-electrode system was used in this experiment. The sample was the working electrode (1 cm^2^), a saturated calomel electrode (SCE) was used as the reference electrode, and a platinum plate was used as the counter electrode. Impedance measurements were performed from 10 mHz to 100 kHz using a 10 mV rms sinusoidal perturbation. 10 experimental points were collected per frequency decade above 66 Hz and 5 experimental points were collected below 66 Hz. The experimental impedance plots were fitted using different equivalent circuits by means of the Zview software. The polarization curves was measured at a scan rate of 2 mV s^−1^. Each polarization curve was measured three times. All the spectra were recorded at open circuit potential. All polarization tests, EIS tests and immersion tests were carried out at room temperature.

## 3. Results and discussion

### 3.1 Film morphology and composition

#### 3.1.1 SEM/EDS analysis

**Fig. 1:**
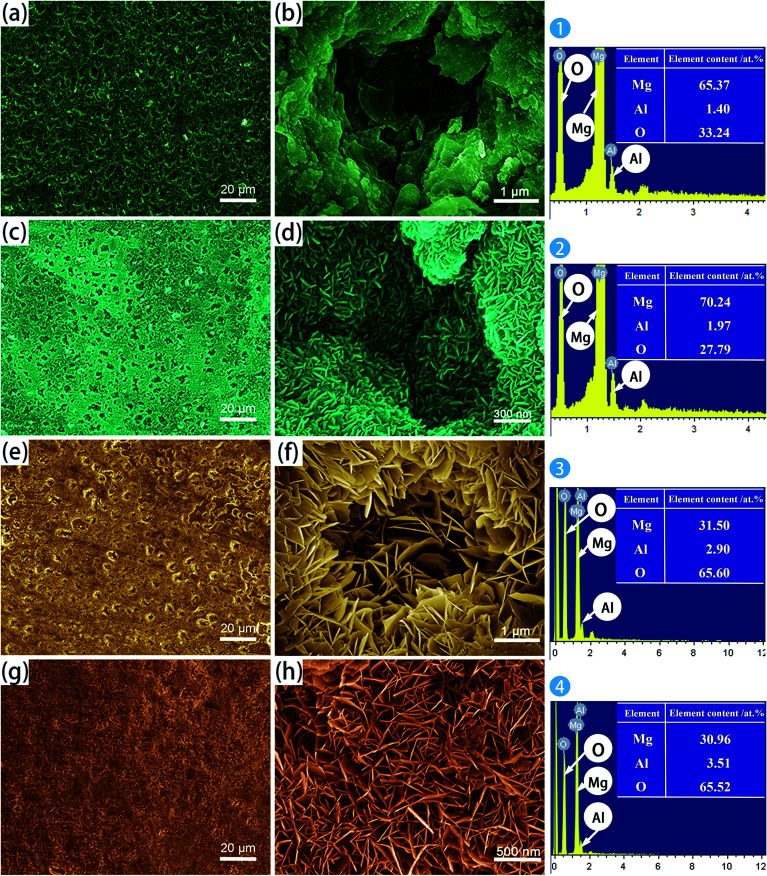
SEM surface micrographs and EDS analysis of: (a and b) A; (c and d) AS; (e and f) A-LDH; (g and h) AS-LDH.

Fig. 1 presents typical SEM micrographs and EDS analyses of the different samples. The morphology of the anodic film (Fig. 1a) reveals a large number of irregular pores, which were distributed throughout the film. Pores with about 4 μm diameter are visible at higher magnification in Fig. 1b. These pores could provide paths for the aggressive media to reach the oxide/metal interface. The EDS analysis shows that the anodic films contain mostly Mg, Al and O, which indicates the presence of suitable ions for the fabrication of the LDHs. Boiling water sealing, sample AS, did not significantly reduce the porosity, as shown in Fig. 1c. However, the higher resolution image of the AS sample shows that the pores were covered by tiny flake-like nanosheets. This phenomenon is not remarkably different from that of an anodized aluminum alloy, which is sealed by boiling water. The mechanism of sealing an anodized aluminum alloy is associated to the formation of boehmite-like products.^33^

After the LDH-sealing, the porosity of the films was reduced but pores still existed as shown in Fig. 1e. The high resolution SEM images indicated that the pores were covered with LDHs. The porosity of the film on the AS-LDH sample was significantly reduced, and the surface of films had become smoother. This could be attributed to the *in situ* fabrication of fine and compact LDH nanosheets. Fig. 1h indicates that the flake-like nanosheets interlace each other and were just like a nest. In addition, EDS data showed that the A-LDH and AS-LDH samples also contained Mg, Al and O. But the ratios of the elements were different from that of A and AS, indicating that phase transitions occurred after the *in situ* fabrication of LDHs. The higher Al/Mg ratio of AS-LDH compared with that of A-LDH indicates the formation of more stable and a larger quantity of interlayer molecules.^34^

#### 3.1.2 XRD analysis

**Fig. 2:**
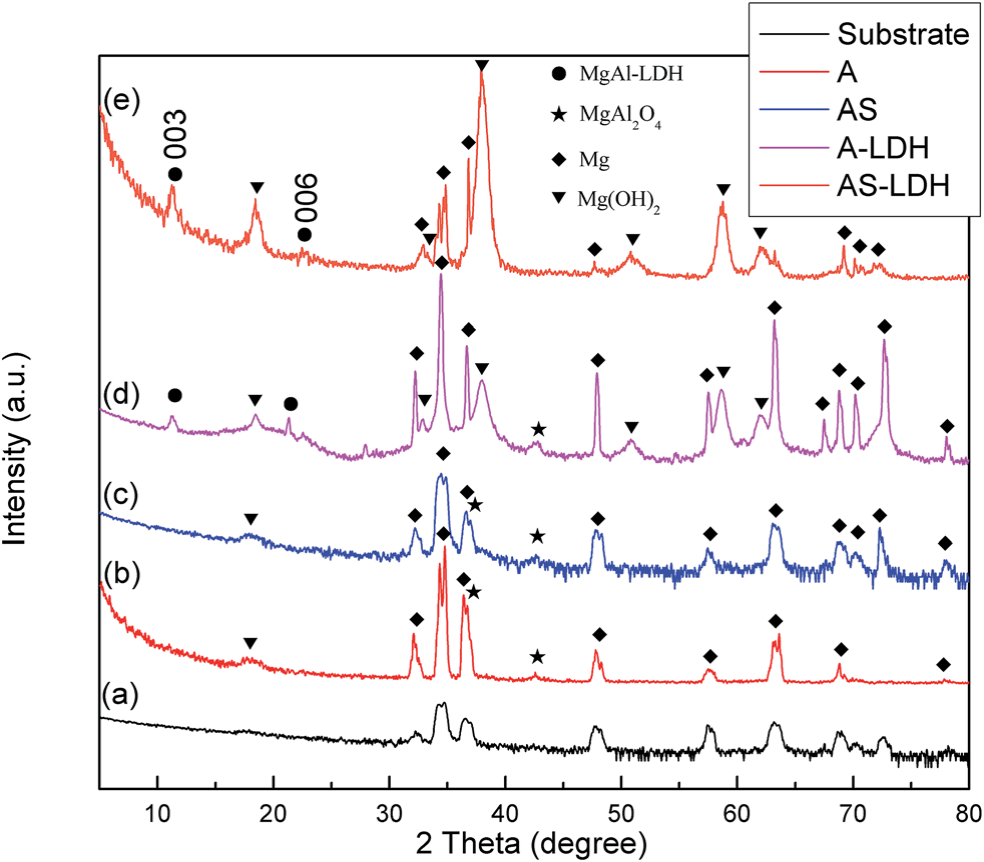
XRD patterns of (a) the substrate; (b) A; (c) AS; (d) A-LDH; (e) AS-LDH.

The XRD patterns of different samples are presented in Fig. 2. The diffraction pattern of the substrate is also presented for comparison purposes. For all samples, the peaks attributable to the Mg alloy substrate are marked with diamonds. A and AS samples were composed of a mixed oxide of Al and Mg (MgAl_2_O_4_) and a small amount of Mg(OH)_2_. This further confirms that Al and Mg oxide/hydroxide was the internal source of cations for the synthesis of the LDHs. Moreover, there was no peak of LDHs in the AS sample, indicating that the tiny nanosheets in Fig. 1d were Mg(OH)_2_. The pattern of A-LDH shows peaks locating at 11.24° and 21.32°, which could be assigned to the 003 and 006 reflections of LDHs. For AS-LDH, peaks at 11.30° and 21.46° also could be assigned to the 003 and 006 reflections of LDHs.^7^ These reflections correspond to a basal spacing of 7.90 and 7.85 Å respectively. These results indicate that A-LDH and AS-LDH possessed a similar interlayer anion. In addition, several peaks which were related to A-LDH and AS-LDH at approximately 2*θ* = 18°, 33°, 38°, 51°, 58° and 62° were assigned to the 001, 100, 101, 102, 110 and 111 diffraction peaks of brucite-type Mg(OH)_2_. This result indicates that the content of Mg(OH)_2_ increased in comparison with that of A and AS. More Mg(OH)_2_ was formed during the formation of the LDHs.

#### 3.1.3 FT-IR analysis

**Fig. 3:**
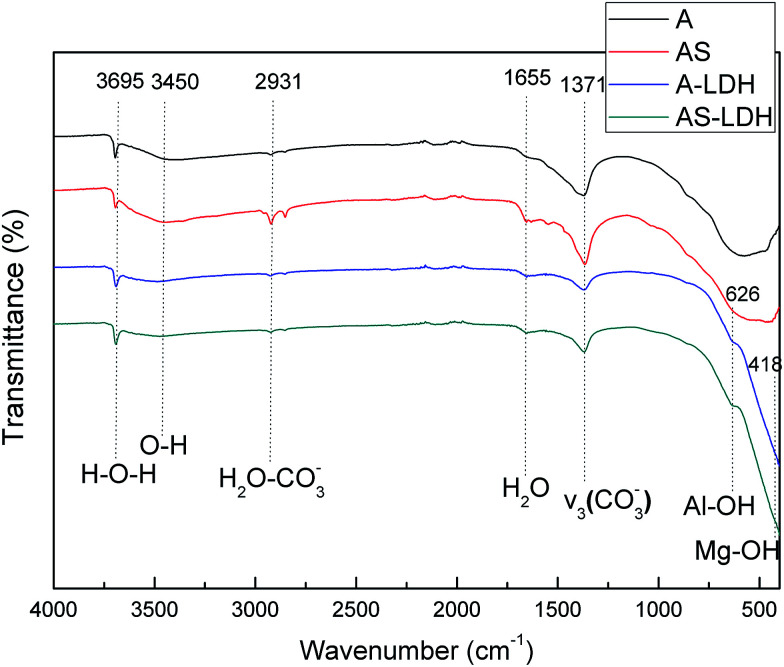
FT-IR spectra of A, AS, A-LDH and AS-LDH.

Fig. 3 presents the FT-IR spectra of the different samples. The absorption bands at approximately 3695, 3450, 1655 cm^−1^ were associated with the stretching vibration of the H-bonds, O–H symmetric contraction and bending vibration of water molecules, respectively. These indicate the presence of surface absorption water or interlayer water.^15^ Some bands for all the samples at approximately 2931 cm^−1^ show the presence of hydrogen bonding between water and CO_3_^2−^. The shoulder bands at 1371 cm^−1^ can be attributed to the symmetric and asymmetric stretching modes of CO_3_^2−^.^1,7^ This result indicates that CO_3_^2−^ is intercalated in the interlayer of LDH sheets. It has been reported the carbonate ions have an exceptionally high affinity to the LDHs.^35^ Moreover, other absorption bands of A-LDH and AS-LDH, which were in the range 800–500 cm^−1^ were mainly due to M–O, M–O–M, and O–M–O lattice vibrations.^36,37^

#### 3.1.4 XPS analysis

**Fig. 4:**
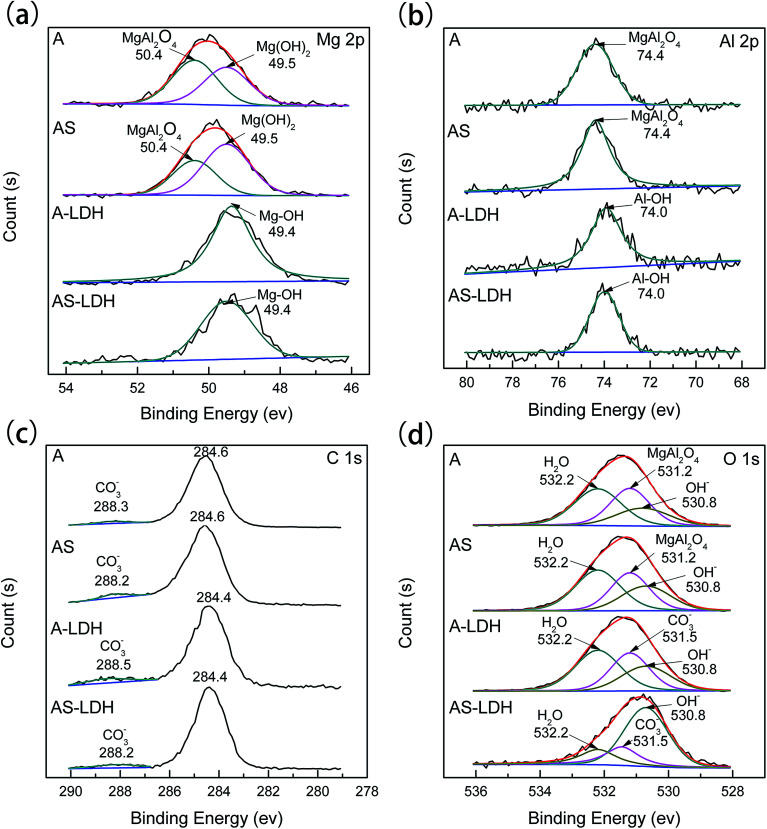
XPS analysis for (a) Mg 2p, (b) Al 2p, (c) C 1s, (d) O 1s spectrum of the different samples.

Fig. 4 shows the XPS spectra for (a) Mg, (b) Al, (c) C, (d) O of the different samples. The peak positions and the full width at half maximum (FWHM) changed after LDH-sealing, especially in the Al and Mg spectrum. These results confirm that the chemical states were different before and after LDH-sealing. Furthermore, deconvolution analyses of the Mg 2p spectra for A and AS show that the peak at 49.5 eV corresponded to Mg(OH)_2_ and the peak at 50.4 eV corresponded to MgAl_2_O_4_.^38^ These results were consistent with that of the XRD analyses. As for A-LDH and AS-LDH, the high energy resolution Mg 2p spectra show a peak at 49.4 eV, which can be attributed to magnesium hydroxyl stretching, which is due to LDHs or Mg(OH)_2_.^32^ The spectra of Al 2p reveal one peak, corresponding to MgAl_2_O_4_ for A and AS whereas aluminium hydroxide for A-LDH and AS-LDH.^39–41^ The high energy resolution C 1s spectra have been fitted with two peaks. One at approximately 284 eV was attributed to the adventitious hydrocarbons from the environment. The other at approximately 288 eV corresponded to CO_3_^2−^.^33^ The spectra of O 1s for A and AS have three peaks at 532.2, 531.2 and 530.8 eV, which were attributed to H_2_O,^37^ MgAl_2_O_4_ (ref. 41) and OH^−^,^42^ respectively. The ratio of peak area of OH^−^ increased after the boiling water sealing treatment. This indicates that more Mg(OH)_2_ formed during boiling water sealing. This result was consistent with that of the SEM image Fig. 2d. The O 1s spectra of A-LDH and AS-LDH were also divided into three peaks of 532.2, 531.5 and 530.8 eV, which corresponded to H_2_O,^37^ CO_3_^2−^ (ref. 33) and OH^−^,^42^ respectively.

#### 3.1.5 Cross-sectional SEM analysis

**Fig. 5:**
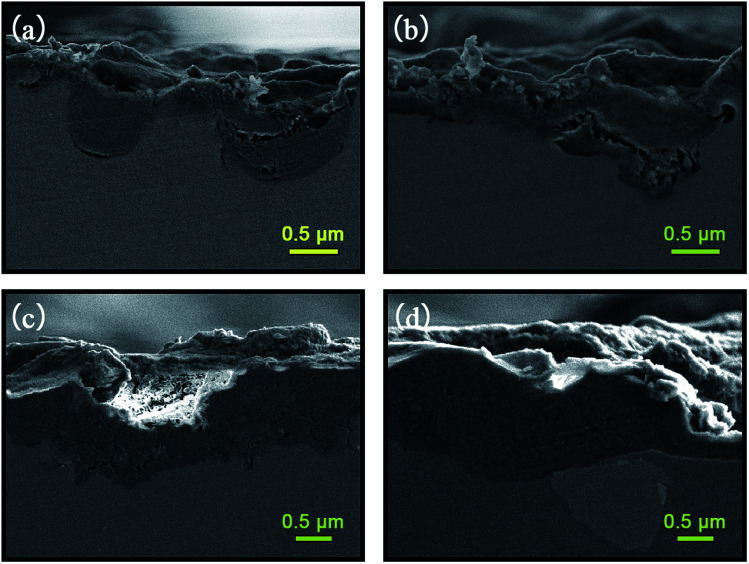
Cross-sectional SEM micrographs of (a) A; (b) AS; (c) A-LDH; (d) AS-LDH.

Fig. 5 shows cross-sectional SEM micrographs of the different samples. In Fig. 5a and b, it can be seen that the anodic layer without and with boiling water sealing have the relatively non-uniform in thickness. Some bulges emerged, due to the concentration of current density around some particular regions. The average thickness of A and AS was 0.8 μm and 1.0 μm, respectively. After fabrication of the LDHs layers on A and AS, the quality of the whole film was obviously improved and the average thickness increased to 2.0 and 1.9 respectively. The increase in thickness may attribute to the outwards growth of LDHs to the film/solution interface.

#### 3.1.6 GDOES analysis

**Fig. 6:**
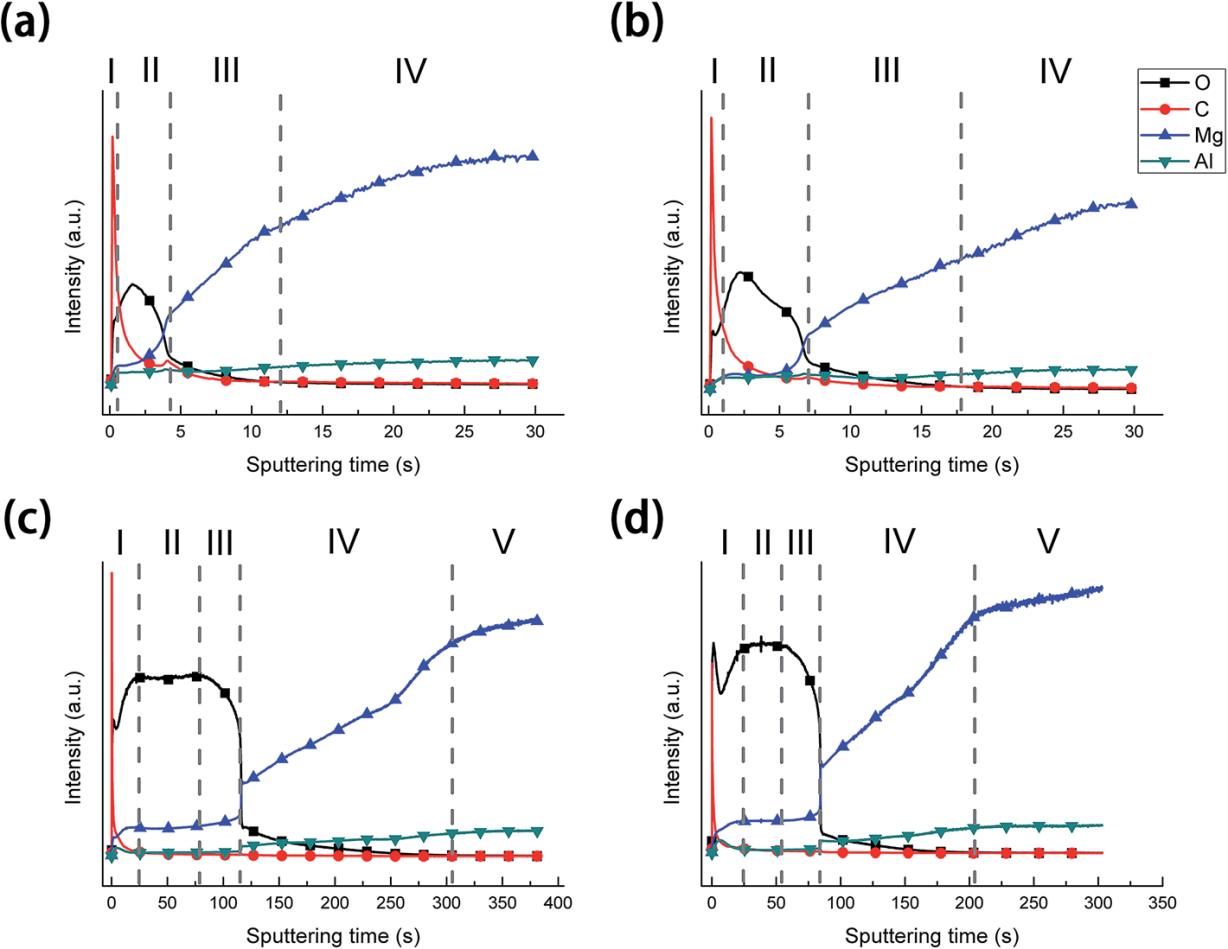
GDOES depth profile of samples: (a) A; (b) AS; (c) A-LDH; (d) AS-LDH.

Depth profiles of the different films are shown in Fig. 6. The curves can be divided into four regions in Fig. 6a and b. In region I, the signal of carbon is high, because of the surface contamination with entrapped and adsorbed CO_2_. In region II, the signal of oxygen was not stable, which was similar to that of previous research^43^ and this result was attributed to the porous structure of anodic films. In region III, the intensities of oxygen and carbon were decreased while the intensity of aluminum was increased. These results confirm that this was the transition from the anodic layer to the substrate during sputtering. The signal between anodic layer and the substrate was not sharp, which may be due to the increasing roughness of the substrate after anodizing. In region IV, the signal reaches the noise level during the sputtering of the substrate. In contrast, the curves of A-LDH and AS-LDH can be divided into five regions. In addition, the sputtering time was significantly longer in comparison with A and AS, attributed to the *in situ* fabrication of the LDHs. At the beginning (region I), the signals can also be attributed to the contamination in the surface of the films. Subsequently, all signals, which reach a plateau, are attributed to LDHs sputtering (region II). The content of oxygen decreased quickly and the contents of magnesium and aluminum gradually increased. These results indicate that this was the transition from LDHs to the anodic layer during sputtering (region III). Because of a very short sputtering time of the anodic layer, there were no obvious sputtering signals for the anodic films. The content of oxygen decreased again due to the simultaneous sputtering of anodic layer and the substrate (region IV). A second plateau of magnesium can be assigned to the sputtering of the substrate (zone IV).

### 3.2 Corrosion resistance of the films

#### 3.2.1 Potentiodynamic electrochemical tests

**Fig. 7:**
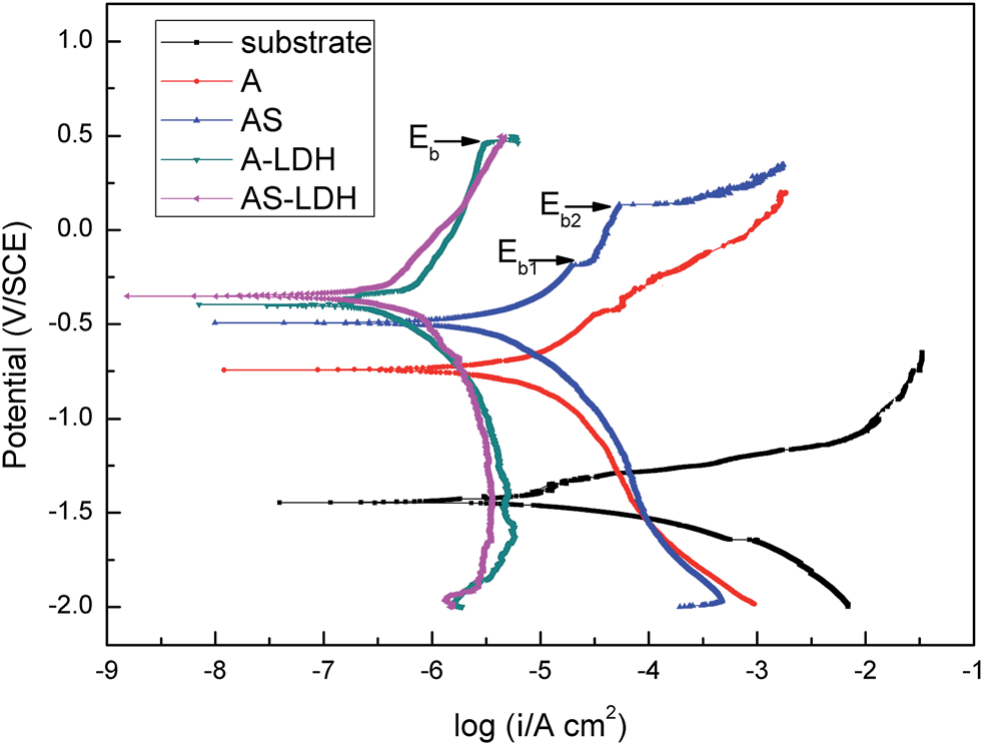
Tafel polarization curves in 3.5 wt% NaCl solution of A, AS, A-LDH and AS-LDH.

Fig. 7 shows the polarization curves of the different samples in 3.5 wt% NaCl solution. The corresponding electrochemical parameters, including corrosion potential (*E*_corr_), corrosion current density (*i*_corr_), anodic Tafel slope (*b*_a_) and cathodic Tafel slope (*b*_c_) are listed in Table 2. Moreover, the corrosion rate (*P*_i_), calculated from *P*_i_ = 22.85*i*_corr_, is also presented in Table 2. The anodic and the cathodic polarization curves were dominated by the Mg dissolution reaction and by the hydrogen evolution reaction respectively. Table 2 indicates that the *E*_corr_ and *i*_corr_ values for the substrate were −1.32 ± 0.31 V_SCE_ and 12.3 ± 2.21 μA cm^−2^. Both the cathodic hydrogen evolution rate and the anodic dissolution rate for all film-coated samples decreased significantly in comparison with that of the substrate. Accordingly, the inhibition effects of all film-coated samples work on both the cathodic hydrogen evolution reaction and the anodic dissolution reaction. After the formation of LDHs, the values of *i*_corr_ decreased by two orders of magnitude compared with that of the substrate. AS-LDH exhibits not only the most positive corrosion potential, but also the lowest corrosion current density. In summary, the corrosion resistance of the magnesium alloy was remarkably improved by LDHs fabricated on the anodized substrate, especially by AS-LDH.

**Table 2:** The corrosion potential (*E*_corr_), corrosion current density (*i*_corr_), the anodic Tafel slopes (*b*_a_), the cathodic Tafel slopes (*b*_c_) derived for the different samples

Sample	*E*_corr_ (V_SCE_)	*i*_corr_ (μA cm^−2^)	Tafel slope (mV dec^−1^)		*P*_i_ (mm per year)
			*b*_a_	*b*_c_	
Substrate	−1.32 ± 0.31	12.3 ± 2.21	153	−76	0.28
A	−0.76 ± 0.02	4.34 ± 0.45	416	−320	0.10
AS	−0.46 ± 0.03	2.85 ± 0.49	434	−437	0.07
A-LDH	−0.36 ± 0.09	0.84 ± 0.32	810	−533	0.02
AS-LDH	−0.29 ± 0.06	0.35 ± 0.03	622	−581	0.01

#### 3.2.2 EIS results

**Fig. 8:**
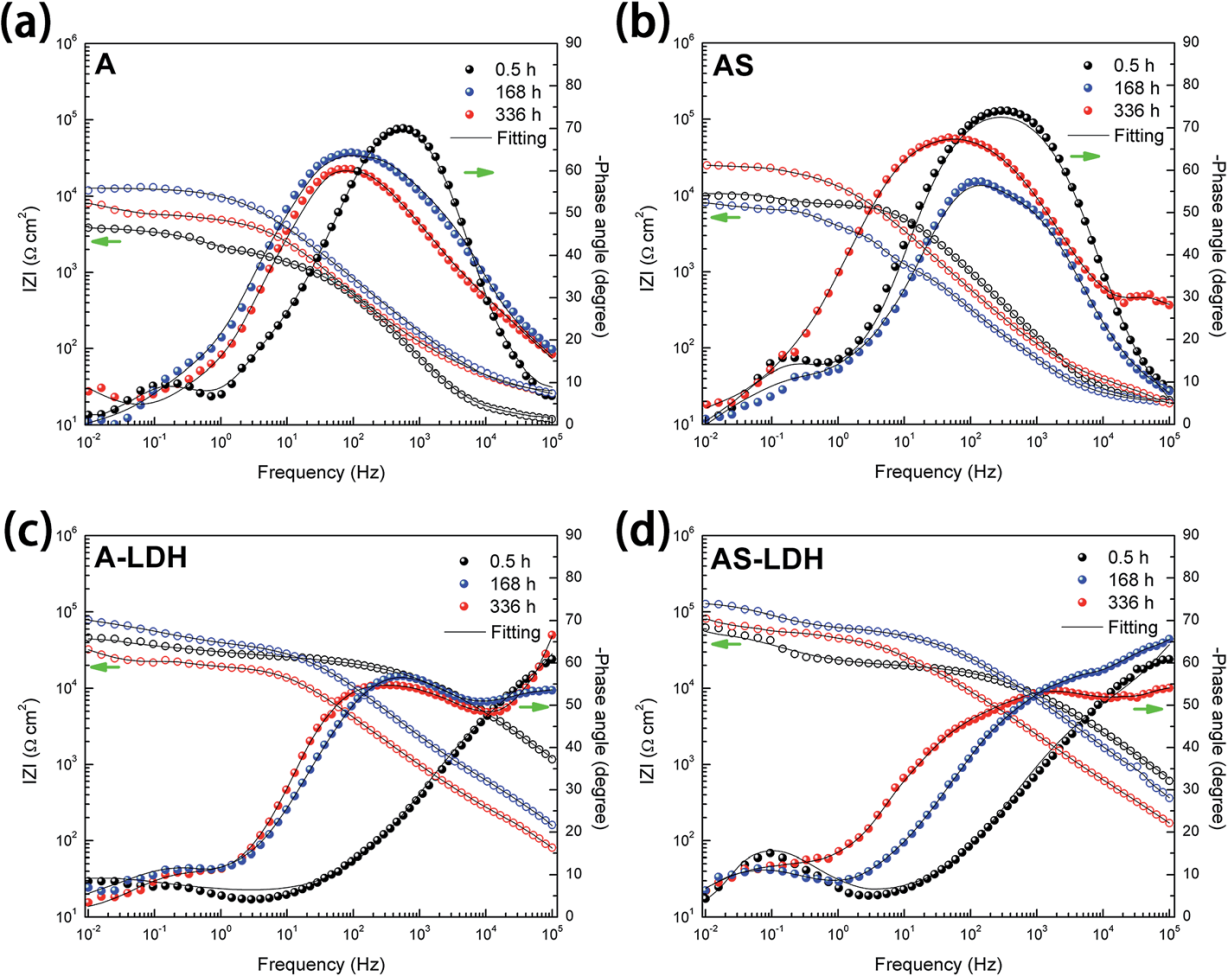
Bode representations of EIS spectra of (a) A; (b) AS; (c) A-LDH; (d) AS-LDH during immersion in 3.5 wt% NaCl solution.

**Fig. 9:**
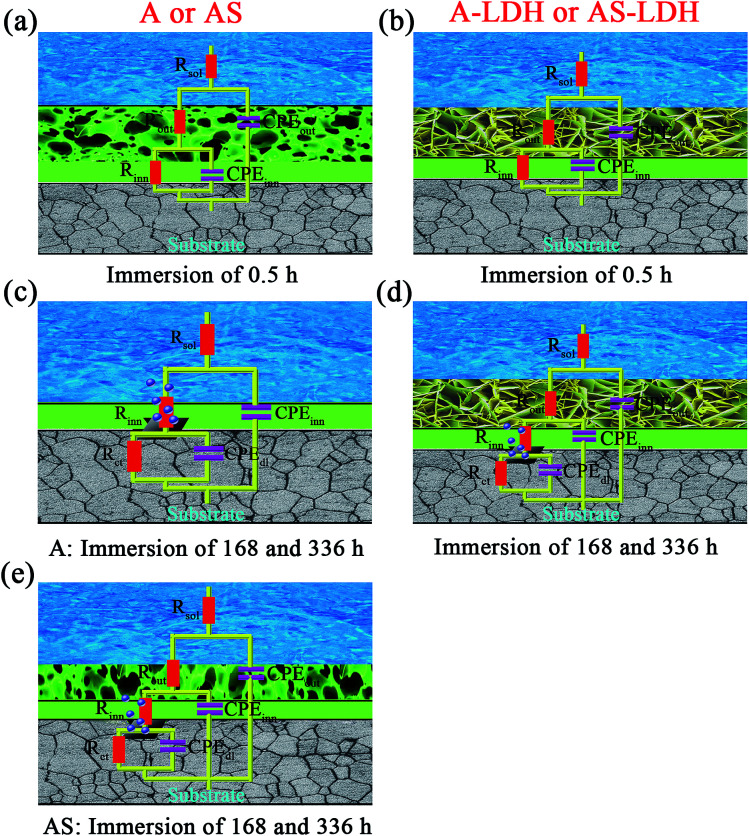
Equivalent circuits used to fit EIS plots and physical models.

In order to study the evolution of different samples, typical Bode plots for different samples after 0.5, 168, 336 h immersion in 3.5 wt% NaCl solution are presented in Fig. 8. At the beginning of immersion (0.5 h), all of the filmed samples present two time constants. One time constant is ascribed to a loose outer layer structure and the other for an inner compact layer structure of the films. The corrosion process of all film-coated samples at this stage can be demonstrated by a physical model and a corresponding equivalent circuit shown in Fig. 9a and b. *R*_sol_ is the resistance of electrolyte; *R*_out_ and *R*_inn_ represent the resistance of loose outer layer and inner compact layer respectively. Instead of the capacitances, the constant phase elements (CPE) are used to demonstrate the non-ideal capacitive behavior of film-coated samples. CPE_out_ and CPE_inn_ describe constant phase elements of outer layer and inner layer, respectively.

For longer immersion time (168 h), the anodized substrate (A) also shows two time constants, while the other samples show the appearance of a third relaxation process. This difference may attribute to outer layer dissolution of the anodic films. As a result, the first time constants can be attribute to an inner layer of the anodic films while the second is associated with the corrosion process. Its physical model and corresponding equivalent circuit are shown in Fig. 9c. Meanwhile, physical models and corresponding equivalent circuits shown in Fig. 9d and e can be used to model the corrosion processes and fit the EIS data for AS, A-LDH and AS-LDH. *R*_ct_ represent the resistance of charge transfer; CPE_dl_ describe constant phase elements of double layer.

After a longer immersion (336 h), AS, A-LDH and AS-LDH all also show three time constants, the first at high frequencies, the second at intermediate frequencies and the third at low frequencies, which are attributed to the LDHs layer (or the outer layer of the anodic films), the inner layer of anodic films and the corrosion process, respectively. This response can also be fitted to the equivalent electric circuit shown in Fig. 9d and e.

**Fig. 10:**
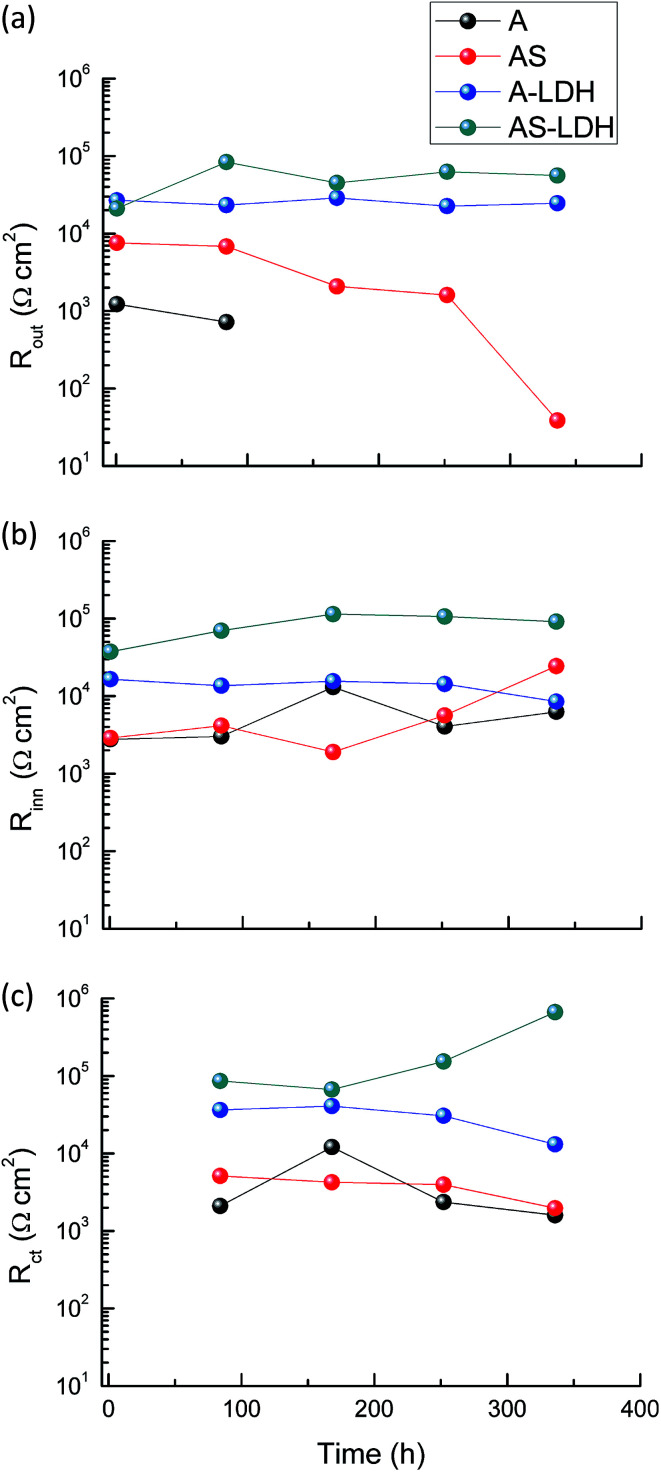
The evolution of (a) *R*_out_, (b) *R*_inn_ and (c) *R*_ct_ as a function of immersion time.

Fig. 10 presents the evolution of the outer layer (*R*_out_), inner layer (*R*_inn_) and charge-transfer resistance (*R*_ct_) for the different samples obtained by fitting the EIS data. Anodic films show resistances of the outer layer for low immersion times (*t* < 84 h), but this response is no longer detected for longer immersion times. Furthermore, this result proves that the anodic films cannot effectively protect the substrate from corrosion in an aggressive environment for a longer period without the sealing treatment. The resistances of the outer layer for the A-LDH and AS-LDH are approximately 40 and 30 kΩ cm^2^, which is 10–40 times larger than that of A and AS. Moreover, the resistance of the outer layer for AS is sharply decreased for longer immersion times (*t* > 252 h), while that of A-LDH and AS-LDH still remain stable. The resistance of the inner layer for AS shows a slightly rising trend with prolonged immersion time, which may be ascribed to the sealing effect of the layered structure Mg(OH)_2_. Interestingly, it was found that *R*_ct_ of AS-LDH showed an increasing trend at longer immersion (*t* > 168 h). In general, the corrosion resistance can be evaluated by the values of *R*_ct_.^26^ Accordingly, the corrosion resistances were ranked as follows: AS-LDH > A-LDH ≫ AS > A.

#### 3.2.3 Immersion tests

**Fig. 11:**
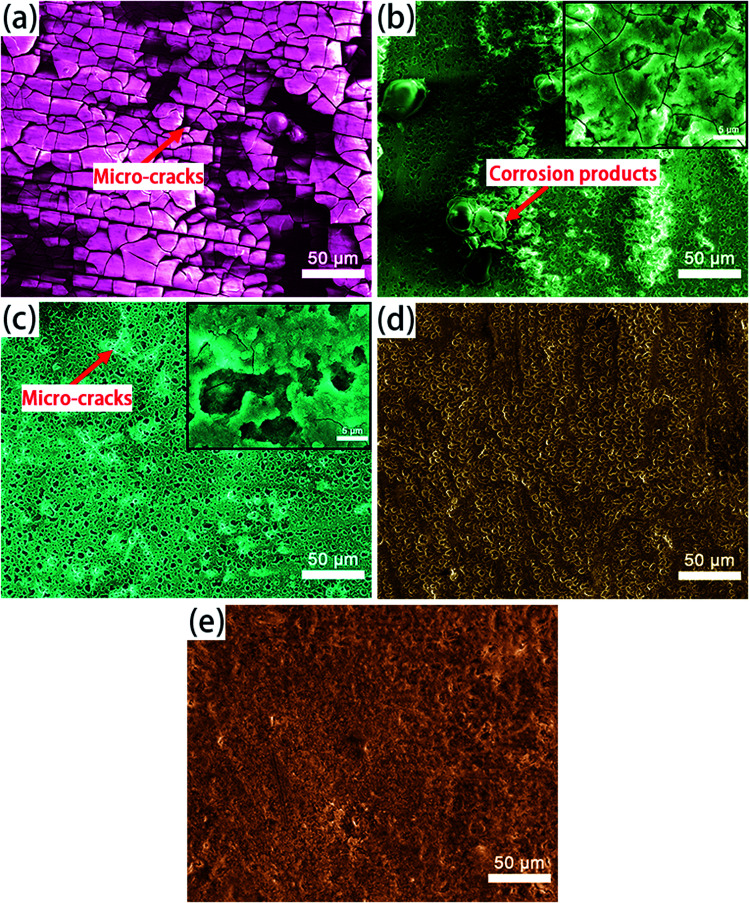
The surface microphotographs of (a) the substrate; (b) A; (c) AS; (d) A-LDH; (e) AS-LDH after 336 h immersion in 3.5 wt% NaCl solution.

Fig. 11 presents the surface morphologies of samples after immersion in 3.5 wt% NaCl solution for 336 h for the different samples. Fig. 11a shows a great many corrosion products distributed on the surface of the substrate everywhere and many micro-cracks. When the anodized substrate was immersed in the NaCl solution, the Cl^−^ cations would get through the pores and reached the films/substrate interface. Subsequently, the substrate Mg was rapidly dissolved into the pore solution and made the pore solution saturated with corrosion products Mg(OH)_2_. The formation of micro-cracks could be attributed to the extrusion stress induced by the accumulation of Mg(OH)_2_.^44^ Therefore, strip-like corrosion products can be observed around the micro-cracks in Fig. 11b. Although the anodized substrate was treated by boiling water for a very short time, there was an obvious difference in comparison with the substrate and anodized substrate without boiling water sealing treatment. Only few micro-cracks were observed in Fig. 11c. The surface of A-LDH was scarcely changed and the pores which were not sealed completely at first were still clearly visible. After 366 h immersion, there were few changes in the surface of AS-LDH because of the strong sealing effect of compact LDHs.

### 3.3 The effect mechanism of boiling water sealing

Based on the above analyses, the effect of boiling water sealing on the growth of LDHs are also discussed as follows. During the anodizing process, metallic Mg dissolves in the aqueous solutions releasing Mg^2+^ cations by an active dissolution reaction with high current density as eqn (1).^45^

1 Mg + H^+^ + H_2_O → Mg^2+^ + OH^−^ + H_2_↑

Simultaneously, Mg^2+^ ions reaching the substrate/solution interface react with AlO_2_^−^, and as a result a mixed oxide of aluminium and magnesium was formed on the metallic substrate.^46^ Khaselev *et al.*^47^ showed that the content of MgAl_2_O_4_ was controlled by the aluminate concentration. It was also reported that elemental aluminium penetrated into the anodic film from the electrolyte as well as from Mg–Al alloy substrate.^46–48^ At the same time, the amorphous Mg(OH)_2_ was formed in the anodic film, as described by eqn (2). That is because eqn (1) leads to the accumulation of OH^−^ ions at the liquid/metal interface and the sample are anodized in an alkaline environment.

2 Mg^2+^ + OH^−^ → Mg(OH)_2_↓

The above reaction likely results in the formation of a higher density of nuclei for Mg(OH)_2_ formation.

During the boiling water sealing process, the anodic layer at high temperatures would have a high kinetic energy and reactivity. As a result, the amorphous Mg(OH)_2_ gradual gather in blocks to form a layered nanostructure in the surface, as shown in Fig. 2d. The presence of a high nuclei density in a given area would hinder the growth of individual particles in 2 dimensions, leading to the formation of a film layer consisting of numerous Mg(OH)_2_ crystallites in the nano-size range. Moreover, the X-ray peaks (Fig. 1) of A and AS for Mg(OH)_2_ show low intensity and broad peaks, which confirmed that Mg(OH)_2_ in this film was present at a low degree of crystallization. The XPS result confirmed that the new Mg(OH)_2_ crystallites were generated at this time. This ultrafine and incomplete nature of such Mg(OH)_2_ deposition provides better protection to the underlying substrate (recall Fig. 11c).

Subsequently, the aluminum atoms that come from the dissolution of the anodic films diffuse into the Mg(OH)_2_ during the LDH-sealing process at the high temperature and pressure. The tetrahedral coordination of aluminum atoms converts into the octahedral one coordinated by hydroxyl groups, resulting in a positive charge on the layers. The carbonate ions, generated from the dissolution of CO_2_ from the air as eqn (3) and (4), are intercalated between the layers in order to maintain charge balance. This result attributes to carbonate ions an exceptionally high affinity to the LDHs. Accordingly, MgAl LDH synthesis could be explained *via*eqn (5).^49^

3 H_2_O + CO_2_ → H^+^ + HCO_3_^−^

4 HCO_3_^−^ → H^+^ + CO_3_^2−^

5 Mg^2+^ + Al(OH)^4−^ + OH^−^ + H_2_O + CO_3_^2−^ → LDH-CO_3_

**Fig. 12:**
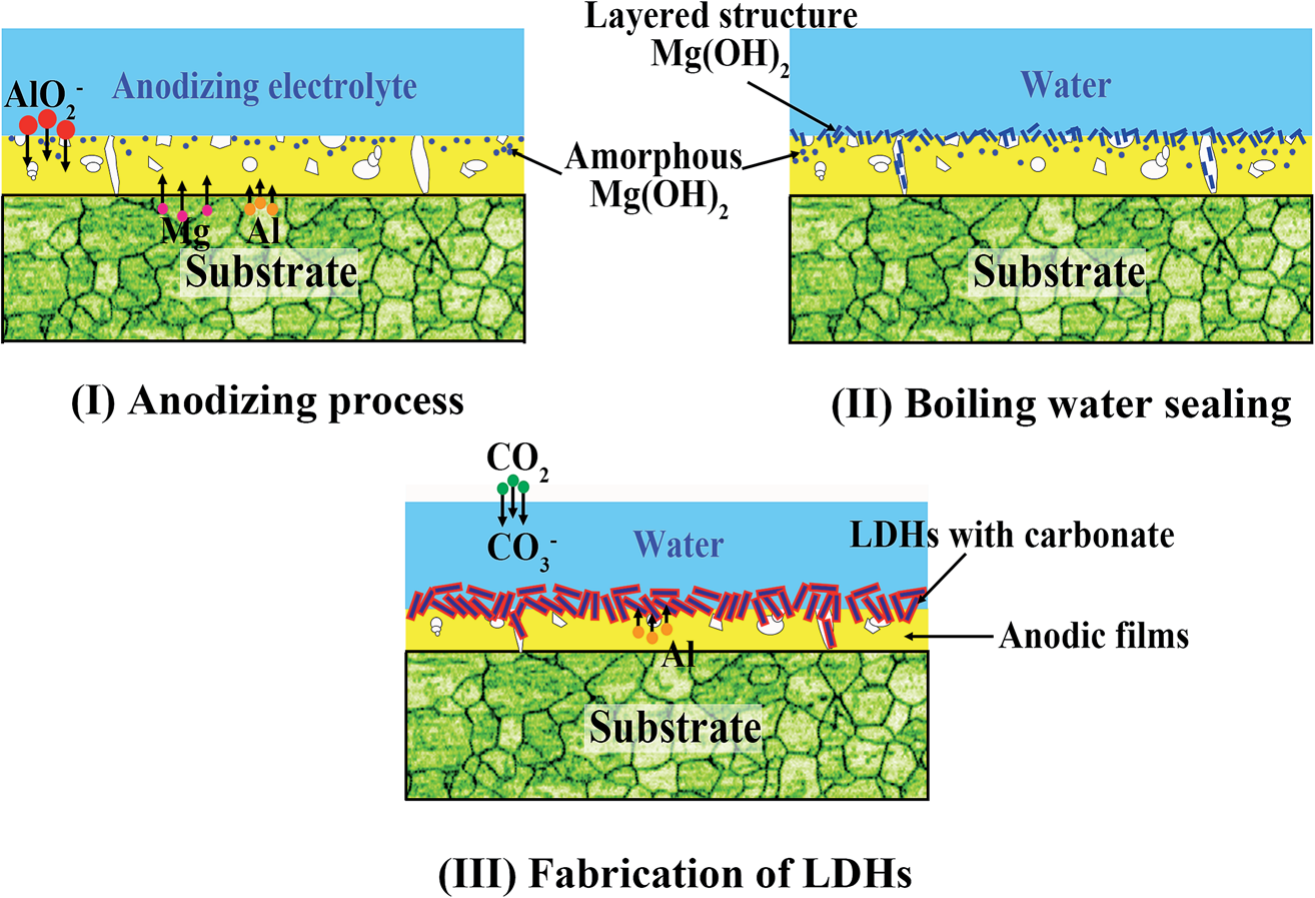
The mechanism model for the effect of boiling water sealing on the growth of LDHs. (I) The anodic film containing MgAl_2_O_4_ and amorphous Mg(OH)_2_ are generated from an active dissolution reaction with high current density. (II) The amorphous Mg(OH)_2_ gradually gathers in blocks to form a layered structure on the surface. (III) The aluminum atoms of the anodic films diffuse into Mg(OH)_2_ during the LDH-sealing process at the high temperature and pressure, which results in formation of LDHs on the anodic film.

This new structure is characteristic of the LDHs. And, this Mg(OH)_2_-based substitution model without formation of polynuclear hydroxo complexes has been reported by Eliseev.^50^ The probable model is proposed, as sketched in Fig. 12. In addition, an other formation model also has been reported. They pointed out that the lamellar γ-AlOOH was formed in the initial stage of hydrolysis. They proposed a “gibbsite-based substitution-filling model” to present the structure of Mg–Al LDHs.^51,52^

## 4. Conclusion

(1) MgAl-LDH with carbonate were successfully fabricated on anodized magnesium alloy of low Al content (AZ31) without introducing any kind of salts, just using anodic films composed of a mixed oxide of aluminium and magnesium as internal source of cations. And, the pH value of the solution was not needed to be adjusted in this method. Moreover, the pores of the anodic films were sealed after the *in situ* fabrication of the LDHs.

(2) Boiling water sealing treatment led to the formation layered structure Mg(OH)_2_, which had a beneficial effect on subsequent growth of LDHs. The morphology of LDHs became fine and compact.

(3) LDHs which were fabricated on the anodized substrate with boiling water sealing treatment showed the best corrosion resistance in comparison with that of the others.

## Conflicts of interest

There are no conflicts to declare.

## Supplementary Material

RA-008-C7RA11683G-s001
