# Supplementary material for: Sealing of anodized magnesium alloy AZ31 with MgAl layered double hydroxides layers
Source: RSC Adv. 2018 Jan 9;8(5):2248–59. doi: 10.1039/c7ra11683g (PMC9077258; doi:10.1039/c7ra11683g)
Supplement: RA-008-C7RA11683G-s001 [file RA-008-C7RA11683G-s001.pdf]

Electronic Supplementary Material (ESI) for RSC Advances.
This journal is © The Royal Society of Chemistry 2017

Sealing of anodized magnesium alloy AZ31 with MgAl layered double hydroxides layers

Gen Zhang[a], Liang Wu[a,b]*, Aitao Tang[a,b]**, Bo Weng[c], Andrej Atrens [d], Shida Ma[a], Lei Liu[a], Fusheng Pan[a,b]

Adhesion testing of **AS-LDH** to the Mg alloy was performed according to ASTM D3359. The cutting tool was fitted with a blade containing five teeth spaced 1.0 mm apart. The cutting tool was used to make the cross-cut pattern at ca. 90◦ angles through the coating. Transparent tape was applied to the cut surface and rubbed with the eraser end of pencil to ensure good contact with the film, and then removed. As can be seen in Fig. S1, there is no delamination or peeling occurred on the cross-cutting surface, indicating that **AS-LDH** has a strong adhesion to the metal substrate.

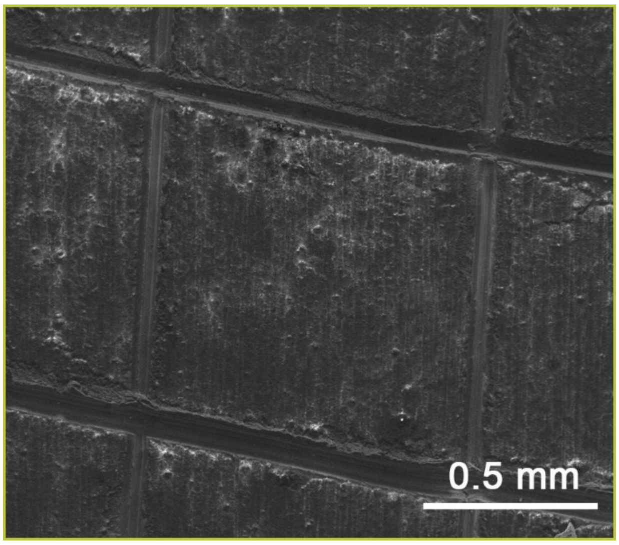

Fig. S1 SEM image of **AS-LDH** on Mg alloy substrate after the cross cut tape test.

Fig. S2 shows the typical Bodes plot for the substarte after 0.5, 168, 336 h immersion in 3.5 wt.% NaCl solution. At the beginning of immersion (0.5 h), it presents two time constants, one corresponds to the natural oxide layer and the other to the

corrosion processes. After a longer immersion (168 and 336 h), the surface was severely corroded leading the formation of a $Mg(OH)_2$ layer. Therefore, R_{ct} show an increased trend. As shown Table S1, it is clearly show that the coated-film samples significantly increase the corrosion resistance of the substrate.

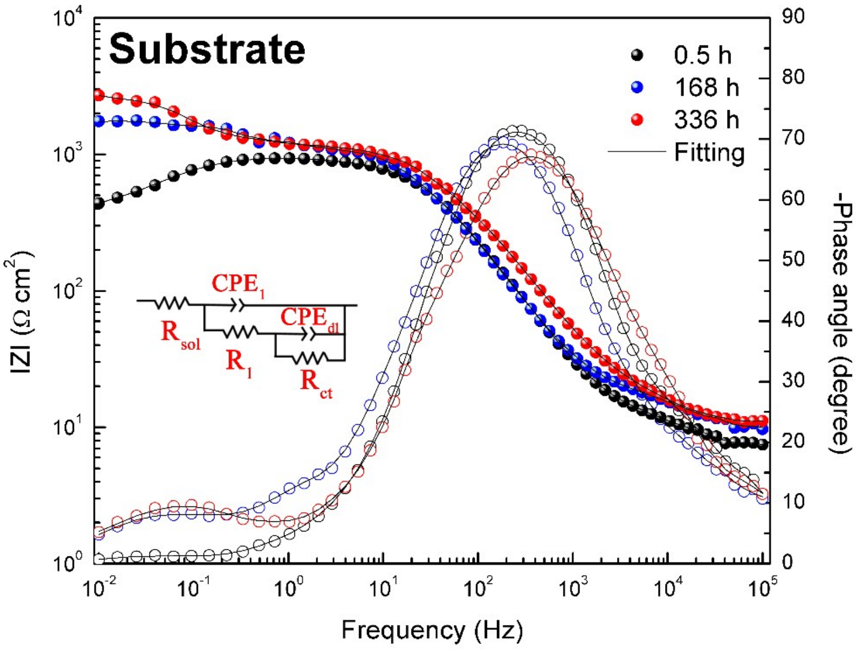

Fig. S2 Bode representations of EIS spectra of the substrate during immersion in 3.5 wt.% NaCl solution.

Table S1

Fitted parameters for EIS spectrum depicted in Fig. S2.

Immersion time	CPE_1 (S s^n cm^{-2})	n_1	R_1 (Ω cm^2)	CPE_{dl} (S s^n cm^{-2})	n_{dl}	R_{ct} (Ω cm^2)	χ^2
0.5 h	1.4×10^{-5}	0.9	1.1×10^{3}	1.4×10^{-8}	0.3	1.0×10^{3}	8.8×10^{-3}
168 h	2.2×10^{-5}	0.8	1.3×10^{3}	9.3×10^{-4}	0.5	1.2×10^{3}	6.7×10^{-3}
336 h	1.4×10^{-5}	0.8	1.2×10^{3}	1.7×10^{-3}	0.3	1.7×10^{3}	3.8×10^{-3}
